# Diminished N1 Auditory Evoked Potentials to Oddball Stimuli in Misophonia Patients

**DOI:** 10.3389/fnbeh.2014.00123

**Published:** 2014-04-09

**Authors:** Arjan Schröder, Rosanne van Diepen, Ali Mazaheri, Diamantis Petropoulos-Petalas, Vicente Soto de Amesti, Nienke Vulink, Damiaan Denys

**Affiliations:** ^1^Department of Psychiatry, Academic Medical Center, University of Amsterdam, Amsterdam, Netherlands; ^2^Netherlands Institute for Neuroscience, an Institute of the Royal Netherlands Academy of Arts and Sciences, Amsterdam, Netherlands

**Keywords:** impulsivity, aggression, sound, misophonia, mismatch negativity, biological markers, auditory event-related potentials

## Abstract

Misophonia (hatred of sound) is a newly defined psychiatric condition in which ordinary human sounds, such as breathing and eating, trigger impulsive aggression. In the current study, we investigated if a dysfunction in the brain’s early auditory processing system could be present in misophonia. We screened 20 patients with misophonia with the diagnostic criteria for misophonia, and 14 matched healthy controls without misophonia, and investigated any potential deficits in auditory processing of misophonia patients using auditory event-related potentials (ERPs) during an oddball task. Subjects watched a neutral silent movie while being presented a regular frequency of beep sounds in which oddball tones of 250 and 4000 Hz were randomly embedded in a stream of repeated 1000 Hz standard tones. We examined the P1, N1, and P2 components locked to the onset of the tones. For misophonia patients, the N1 peak evoked by the oddball tones had smaller mean peak amplitude than the control group. However, no significant differences were found in P1 and P2 components evoked by the oddball tones. There were no significant differences between the misophonia patients and their controls in any of the ERP components to the standard tones. The diminished N1 component to oddball tones in misophonia patients suggests an underlying neurobiological deficit in misophonia patients. This reduction might reflect a basic impairment in auditory processing in misophonia patients.

## Introduction

Misophonia is a newly defined psychiatric condition, which is characterized by the hatred of ordinary human sounds (Hadjipavlou et al., 2008; Schwartz et al., 2011; Edelstein et al., 2013; Ferreira and Harrison, 2013; Neal and Cavanna, 2013; Schröder et al., 2013). The central hallmark of misophonia is an aggressive impulse automatically triggered by sounds, such as breathing, chewing, and eating. To date, there has been no neurophysiological marker linked with this disorder. Such a marker could potentially benefit the recognition of misophonia patients and give directions to further neurophysiological research in this domain.

The underlying causes of misophonia are unknown. Patients usually report normal hearing and standard hearing tests do not reveal any audiological deficits (Edelstein et al., 2013; Schröder et al., 2013). Therefore, our aim was to explore if the pathophysiology of this disorder manifested itself in some dysfunction of the auditory processing system. This was carried out by examining differences in specific components of the auditory event-related potentials (ERPs) between misophonia patients and controls, elicited by pure tones in an oddball paradigm.

Early sensory components evoked by auditory stimulation include a positive peak around 50 ms (P50 or P1), a negative peak around 100 ms (N100 or N1), and a positive peak around 200 ms (P200 or P2). To date, there have been a number of studies examining anomalies in these components in various psychiatric disorders such as schizophrenia, bipolar disorder, and posttraumatic stress-disorder (PTSD) (O’Donnell et al., 1994; Javitt et al., 2000; Kayser et al., 2001; Salisbury et al., 2010; Javanbakht et al., 2011).

The P1 is associated with pre-attentive orienting toward new sounds and is not yet affected by attention (Picton and Hillyard, 1974; Pratt et al., 2008). Sensory gating (the suppression of responding to irrelevant stimuli) can be assessed by examining P1 suppression during repetitive presentation of an auditory-stimulus (Luck and Kappenman, 2012). Its amplitude is found to be altered in various disorders such as autism, Alzheimer’s disease, and attention-deficit/hyperactivity disorder (Buchwald et al., 1989, 1992; Kemner et al., 1996).

The N1 peak is linked to early attention (Näätänen, 1992; Rinne et al., 2006). It has been suggested to signal the detection of abrupt changes in sensory input, which enables us to focus on events that are potentially informative (Friston, 2005; Winkler, 2007; Todd et al., 2012). The N1 is commonly assessed in an “oddball” paradigm (Näätänen and Picton, 1987). In such paradigm, participants are presented repetitive sounds (“standard”) with randomly occurring rare deviant sounds (“oddballs”), often while watching a silent movie. Attenuated N1 responses have been found in various studies of schizophrenia, a disorder in which notable audiological symptoms, i.e., acoustic hallucinations and delusions, are present. In these studies, N1 peaks were reduced in chronic schizophrenics, first-hospitalized patients, and twins of schizophrenics (Salisbury et al., 2010). N1 findings in PTSD are inconsistent with some studies reporting increased N1 amplitude and others decreased amplitude. Peak latency increments were also found but not in all studies (Javanbakht et al., 2011). Interestingly, in antisocial personality disorder, an impaired N1 was related to increased impulsivity (Lijffijt et al., 2012). Decreased N1 function was also found in cocaine abuse and bipolar disorder (Boutros et al., 2006; Lijffijt et al., 2009).

The P2 peak is an endogenous evoked component and appears to be involved in early allocation of attention and initial conscious awareness (Näätänen, 1992). Less research has been focused on the P2 peak. Reduced P2 has been found in chronic and in first-hospitalized schizophrenic patients (O’Donnell et al., 1994; Salisbury et al., 2010). In PTSD studies, the results are ambiguous with both increased and decreased P2 amplitudes being reported (Javanbakht et al., 2011).

In our present study, we investigated early processing of auditory information using a non-attending oddball paradigm. We focused our analysis on any differences in the P1, N1, and P2 components of the evoked potentials between patients diagnosed with misophonia, and matched healthy controls. While the exogenously generated P1 component could provide information about sensory gating, a P2 difference would point more toward attention-related malfunctioning. Because the N1 is considered the most stable ERP component, this could be a reliable marker of pathology. We believe that any difference between the auditory evoked responses of misophonia patients and controls could reflect an anomaly in the way that these patients filter novel information in the auditory environment.

## Materials and Methods

### Participants

Twenty patients with misophonia (males = 11, females = 9, aged 20–55 years, *M* = 35.9 years, SD = 10.6 years) were screened with the diagnostic criteria for misophonia and severity was assessed with the Amsterdam Misophonia Scale (AMisoS) (Schröder et al., 2013). All patients were tested for auditory impairments (McArdle and Hnath-Chisolm, 2009; Schlauch and Nelson, 2009). Psychiatric status was evaluated using the Symptom Checklist (SCL-90) (Derogatis et al., 1973), Hamilton Depression Rating Scale (HAM-D) (Hamilton, 1960), and Hamilton Anxiety Rating Scale (HAM-A) (Hamilton, 1959).

Fourteen healthy controls (males = 11, females = 3), matched for demographical characteristics, were recruited on the absence of any misophonic symptoms or psychiatric comorbidity and tested. The age ranged between 23 and 55 years (*M* = 32.4 years, SD = 9.0 years).

Subjects were tested for hearing deficits using standard hearing tests (tone and speech audiogram and loudness discomfort levels) and no deficits were found. Complementarily, both groups filled out the Profile of Mood States (POMS) – short form, which assessed arousal level and mood on five subscales (Tension–Anxiety, Depression–Dejection, Anger–Hostility, Fatigue–Inertia, and Vigor–Activity). The overall assessment of the current emotional state – the total mood disturbance (TMD) score – was calculated by adding up the first four negative subscale scores and subtracting the Vigor–Activity score (McNair et al., 1992; Curran et al., 1995). A higher TMD score denotes a more negative affective state.

The characteristics of both groups are provided in Table 1. All participants gave written informed consent and received financial compensation for their travel expenses, but no further compensation was offered for participating in the study. The study was carried out in accordance with the Declaration of Helsinki and was approved by the local medical ethics committee of the Academic Medical Center of Amsterdam.

**Table 1:** **Clinical and demographic characteristics of the study sample**.

	Misophonia patients (*N* = 20)	Controls (*N* = 14)
Age (years)^a^	35.9 (10.6)	32.4 (9.0)
Gender (male/female)^b^	11/9	11/3
Comorbidity	Remitted depressive disorder 1	–
	Remitted GAD 1	
	ADHD 1	
Age of onset	12.0 (4.9)	–
Medication use	Antidepressants 5^d^	Anxiolytics 1^e^
	Anxiolytics 1^e^	
	Stimulants 1^f^	
**QUESTIONNAIRES**		
POMS^c^	1.0 (10.3)	−7.1 (4.7)
HAM-A	11.5 (9.3)	–
HAM-D	8.6 (7.7)	–
SCL90	150.6 (44.0)	–
AMisoS	14.3 (3.6)	–

*^a^Mann–Whitney test: *p* = 0.323*.

*^b^χ^2^ test, groups are not significantly different in male/female ratio, *p* = 0.157*.

*^c^Mann–Whitney test, *p* = 0.004. Baseline emotional state between groups is significantly different*.

*POMS, Profile of Mood Scale [Total Mood Disturbance (TMD) scores]; HAM-A = Hamilton Anxiety Scale; HAM-D = Hamilton Depression Rating Scale; SCL90 = 90 items Symptom Checklist; AMisoS = Amsterdam Misophonia Scale (concept scale for symptom severity; 0–4 subclinical misophonic symptoms, 5–9 mild, 10–14 moderate, 15–19 severe, 20–24 extreme)*.

*^d^Antidepressants: 2 patients – venlafaxine, 1 patient – sertraline, 1 patient – citalopram, 1 patient – fluoxetine*.

*^e^Anxiolytics: oxazepam*.

*^f^Stimulants: methylphenidate*.

### Auditory paradigm

The participants were presented with a pseudorandomized sequence of 840 tone stimuli (Presentation 11.3, Neurobehavioral Systems Inc., Albany, CA, USA) administered through Philips SHS3201/28 headphones. The standard tones (80%) had a frequency of 1000 Hz. A deviant tone that was lower than the standard tone (250 Hz) and a tone that was higher than the standard (4000 Hz) were added to the sequence. Both deviants were presented in 10% of trials and were never presented successively.

The auditory stimuli had a duration of 200 ms (including 10 ms rise and fall times shaped by a Blackman window), while the inter-stimulus interval was 650 ms. During the presentation of the tones, the participants watched a neutral silent movie with subtitles. They were instructed to ignore the tones.

### Data acquisition

EEG data were acquired using a WaveGuard 10–5 cap system developed by ANT, with 64-Ag/AgCl electrodes, spanning from frontal, temporal, central, and occipital scalp sites. The EEG was sampled at 512 Hz with an online average reference and then subsequently imported into MATLAB for all further off-line analyses. The electrooculogram (EOG) was recorded between supra- and infra-orbital sites around the left eye for vertical movement (blinks), and outer acanthi of the left and right eyes for possible side-eye movements.

### EEG preprocessing

Data analysis was completed using EEGLAB^1^ and Fieldtrip software packages^2^ along with in-house scripts. Data were high-pass filtered at 0.5 Hz using a non-causal FIR filter (“fir1” in EEGLAB). Trials containing artifacts (e.g., eye movements, blinks, muscle potentials) were removed from the EEG using the default automatic-reject routines in EEGLAB. Independent component analysis was used to remove any eye movements not rejected by the semiautomatic routines (Jung et al., 2001).

### ERP analysis

The auditory-stimulus locked ERP data were low-pass filtered at 30 Hz using a two-pass Butterworth IIR filter (default option Fieldtrip) and averaged with the sweep beginning 200 ms before the stimuli and lasting until 450 ms after stimulus onset. The ERPs were baseline corrected using the mean time 150 ms prior to stimulus onset.

The average peak amplitude and peak latency of the P1, N1, and P2 were computed per subject and compared between the misophonia patient group and healthy controls. The time interval for determining the mean peak amplitude and latency were chosen based on looking at the grand-averaged data.

The difference in the P1, N1, and P2 response between misophonia patients and controls was assessed separately for the standard and deviant tones. This was due to the assumption that different processes likely take place after presentation of a frequent and infrequent tone, which could be affected differently in patients. Moreover, separating analyses for standard and deviant tones could also circumvent the potential problem of comparing conditions with a difference in signal-to-noise ratio, arising from the difference in amount of trials between the standard and deviant condition (Salisbury et al., 2010).

For the deviant tones, peak latency of the P1 was defined as the most positive deflection occurring between 50 and 100 ms post-stimulus onset in electrodes Fz and FCz. N1 peak latency was defined as the most negative deflection in Fz and FCz occurring between 100 and 200 ms, and the P2 was defined as the most positive deflection in Cz and FCz between 200 and 300 ms.

For the standard tones, evoked responses were different in timing and therefore different time-windows were used for the N1 (120–160 ms) and P2 (160–220 ms). Channel selection was based on maximal amplitude of the grand average.

The mean amplitudes for the P1, N1, and P2/3 were obtained by averaging values of abovementioned channels within the predefined intervals.

An independent *t*-test was performed to test for a difference in responses elicited by the standard tones. Two-way repeated-measures analysis of variance (rANOVA) was used to test for a difference in response after presentation of deviant tones, with tone (low deviant, high deviant) as within-subject variable and group (control, patient) as between-subject variable. Statistics were performed using SPSS, attaining a threshold of *p* ≤ 0.05. For clarity reasons, only significant effects will be reported in the Section “Results.” Results of insignificant effects are presented in Table 3.

## Results

### Study participants

Clinical characteristics are presented in Table 1. There was no significant difference in age between the patient group and the control group (*p* = =0.323). Also, the male/female ratio was not significantly different [*X*^2^(1, *N* = 34) = 2.004, *p* = 0.157]. Compared to controls, misophonia patients reported a significantly higher baseline emotional state [*t*(28.39) = 3.11, *p* = =0.004], measured by the TMD score on the POMS.

### Event-related potentials

**Figure 1:**
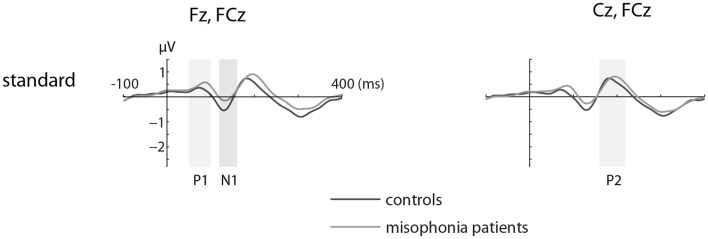
**The grand-average ERP waveforms of controls (*n* = 14) and misophonia patients (*n* = 20) for standard tones**. Standard tones elicited equal responses for patients and controls when compared for average amplitude and peak latency of the P1, N1, and P2. Shaded areas represent time intervals of interest.

**Figure 2:**
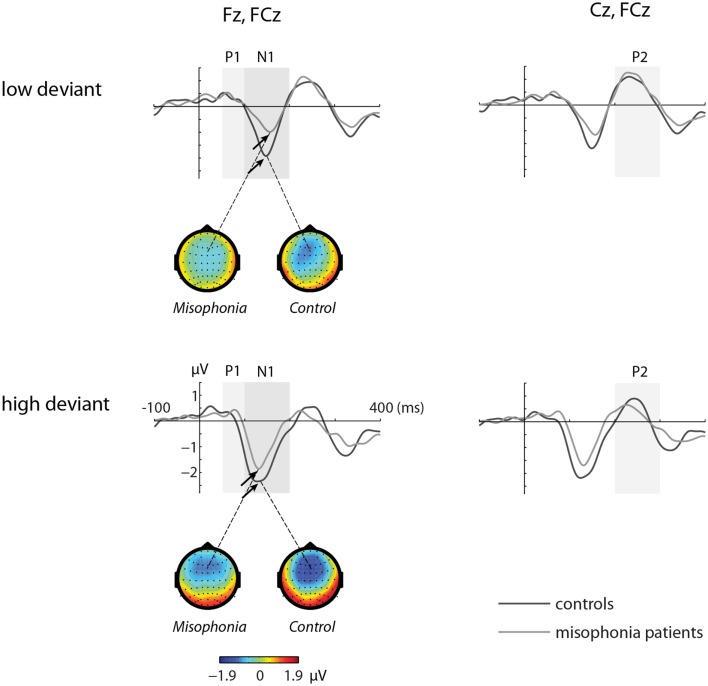
**The grand-average ERP waveforms of controls (*n* = 14) and misophonia patients (*n* = 20) for deviant tones (lower and higher than standard tone)**. Shaded areas represent time intervals of interest. Deviant tones elicited a diminished N1 response in misophonia patients compared to controls. Topographies of the N1 are shown for the low and high deviant tone.

Figures 1 and 2 show the grand-average ERP evoked by standard (Figure 1) and deviant tones (Figure 2) in the same electrodes used for the statistical analyses. The shaded areas represent time intervals of interest.

### Deviant tones elicited a diminished N1 in misophonia patients

The deviant tones evoked a smaller N1 component in the misophonia patients than in the control group [−0.711 vs. −1.277 μV, *F*(1,32) = 5.608, *p* = 0.024]. However, the peak latency of the deviant evoked N1 was not different between the misophonia and the healthy control group. We found no differences in the P1, P2 average amplitude or peak latencies between patients and controls (see Tables 2 and 3). Also no interactions between group and deviant tones were found.

**Table 2:** **Group averages of mean amplitudes (in bicronvolt) and peak latencies (in milliseconds) for the different tones (standard, low deviant, high deviant) and components (P1, N1, and P2)**.

	P1		N1		P2	
	Control	Patient	Control	Patient	Control	Patient
**AVERAGE AMPLITUDE**						
Standard	0.281	0.463	0.290	0.031	0.552	0.635
Low deviant	0.324	0.337	−0.941	−0.468	0.705	0.812
High deviant	−0.080	0.219	−1.614	−0.95	0.357	0.299
**PEAK LATENCY**						
Standard	77	84	135	135	187	197
Low deviant	68	73	150	152	242	240
High deviant	65	75	133	139	246	232

**Table 3:** ***p*-Values for rANOVA and *t*-tests on average amplitude and peak latencies**.

	P1	N1	P2
**AVERAGE AMPLITUDE**			
Deviants (ANOVA)			
Main effect tone	**0.035**	**0.001**	**0.007**
Main effect group	0.245	**0.024**	0.916
Interaction	0.236	0.539	0.583
Standard (*t*-test)
	0.072	0.274	0.710
**PEAK LATENCIES**			
Deviants (ANOVA)
Main effect tone	0.938	**0.0001**	0.761
Main effect group	0.122	0.418	0.210
Interaction	0.398	0.550	0.336
Standard (*t*-test)
	0.148	0.884	0.096

*Significant values are printed in bold*.

A main effect of tone was present for the P1, N1, and P2 average amplitudes. The low deviant tone elicited a larger P1 than the high deviant tone [0.332 vs. 0.096 μV, *F*(1,32) = 4.840, *p* = 0.035]. In contrast, the lower deviant tone evoked a smaller N1 than the high tone deviant [−0.662 vs. −1.226 μV, *F*(1,32) = 5.537, *p* = 0.001]. The P2 was larger after presentation of the lower deviant than the high deviant tone [0.768 vs. 0.323 μV, *F*(1,32) = 8.457, *p* = 0.007].

Finally, the peak latency of the N1 response was different for the two deviant tones, such that the high tone showed an earlier peak compared to the low tone [136 vs. 151 ms, *F*(1,32) = 20.097, *p* = 0.0001].

### Standard tones elicited a similar auditory evoked response for patients and controls

We found no differences in the average amplitude and peak latency of the P1, N1, or P2 responses elicited by the standard stimuli between the misophonia and control group.

## Discussion

We found that the mean amplitude of the auditory N1 was significantly diminished in misophonia patients compared to healthy controls. This attenuation suggests a deficit in auditory information processing at a low-level in misophonia patients.

One possible explanation of the smaller N1 peak in misophonia patients in our study might be the difference in the clinical characteristics of the two groups. The most notable difference was the TMD scores on the POMS (Table 1). Misophonia patients had a significantly higher TMD than the controls. An increased TMD could reflect a state of general hyperarousal in misophonia patients. Due to this hyperarousal or general irritability, misophonia patients might not have attended to the sounds as much as the controls. The link between general hyperarousal and misophonia has also been discussed by Edelstein et al. (2013) who found a significantly higher skin conductance response in the misophonia group triggered by various visual and auditory aversive stimuli. Both misophonia patients and controls found similar stimuli to be aversive and non-aversive but on a different level. They therefore raised the possibility that misophonia patients are merely at the tail end of the distribution.

Another explanation could be that difference in N1 peak amplitude between the misophonia group and the control group is due to some other psychiatric comorbidity or the use of psychotropic medication. However, we believe that it is very unlikely that these differences can be explained by comorbidity because in the misophonia group only one patient had a current psychiatric comorbidity, which was attention-deficit hyperactivity disorder (ADHD). Nevertheless, the confounding effect of psychotropics, especially antidepressants, on N1 responses in misophonia patients cannot completely be ruled out. However, previous research investigating medication effects on the N1 indicate that this is unlikely (Salisbury et al., 2010).

We concede that our current findings cannot easily be linked to two fundamental issues underlying misophonia symptomology: first, why do human sounds – and not inanimate, i.e., environmental sounds – evoke misophonic symptoms? And second, why do these sounds trigger aggression (Schröder et al., 2013)?

We conjecture that the first issue could be related to the existence of two separate neural systems for processing human and non-human sounds (Pizzamiglio et al., 2005; Engel et al., 2009). Possibly, in misophonia it is only the human sound-processing network that is affected. This, however, does not fully explain the arousal described by a few misophonia patients caused by inanimate – environmental – sounds (Edelstein et al., 2013), or why we observed a lower N1.

The second question might be understood through literature on obsessive–compulsive personality disorder (OCPD). OCPD has a very high comorbidity rate in patients with misophonia of 52.4% (Schröder et al., 2013). Core symptoms of OCPD include cognitive inflexibility and high levels of moralism, which can result in criticism of other people’s behavior (American Psychiatric Association, 2000). Violation of social norms – both intentional and unintentional – have been associated with left orbitofrontal cortex (OFC) activation (Berthoz et al., 2002). Therefore, OFC involvement in the emergence of misophonia symptoms could exist. This could then also partly explain the differences, described by some misophonia patients, between their aggressive reactions to sounds made by, e.g., babies or elderly demented, which usually do not trigger much aggression, and to those of other adults, which do. The difference might lie in the level that the misophonia patients assess the accountability and deliberateness of the sound source.

## Conclusion

This is the first study investigating the underlying neurobiological mechanisms of misophonia. We found that it was possible to distinguish misophonia patients from healthy controls by using a simple auditory oddball paradigm. We conclude that a lower than normal N1 response could be a neurophysiological marker for misophonia. However, it still remains to be investigated if this diminished N1 is a characteristic of general psychiatric psychopathology or a distinctive characteristic for misophonia. Moreover, it is unclear whether the underlying deficit in misophonia is due to altered auditory perception, an inadequate processing of auditory stimuli, or a higher order dysfunction of cortical control related to impulsivity. Thus, we believe further research should therefore aim at delineating misophonia from other psychiatric disorders and elucidate the neural interactions directly correlating with the symptomology of misophonia.

## Conflict of Interest Statement

The authors declare that the research was conducted in the absence of any commercial or financial relationships that could be construed as a potential conflict of interest.

## Footnotes

^1^http://sccn.ucsd.edu/eeglab/

^2^http://www.ru.nl/fcdonders/fieldtrip/
